# Chemotherapy-induced nausea and vomiting in patients with breast cancer: a prospective cohort study

**DOI:** 10.1007/s12282-019-01001-1

**Published:** 2019-08-12

**Authors:** Yoichi Naito, Yuichiro Kai, Takashi Ishikawa, Tomoyuki Fujita, Kanou Uehara, Hiroyoshi Doihara, Shinya Tokunaga, Mototsugu Shimokawa, Yoshinori Ito, Toshiaki Saeki

**Affiliations:** 1 Department of Breast and Medical Oncology, National Cancer Center Hospital East, 6-5-1 Kashiwanoha, Kashiwa, Chiba 277-8577 Japan; 2 Department of Developmental Therapeutics, National Cancer Center Hospital East, Kashiwa, Japan; 3 Ueo Breast Cancer Hospital, Oita, Japan; 4 Department of Breast and Thyroid Surgery, Yokohama City University Medical Center, Yokohama, Kanagawa Japan; 5 Department of Breast Surgery, Tokyo Medical University Ibaraki Medical Center, Ami, Ibaraki Japan; 6 Department of Surgery, Nahanishi Clinic, Naha, Okinawa Japan; 7 Department of Breast and Endocrinological Surgery, Okayama University Hospital, Okayama, Japan; 8 Department of Medical Oncology, Osaka City General Hospital, Osaka, Japan; 9 Center of Clinical Research, National Kyushu Cancer Center, Fukuoka, Japan; 10 Department of Breast Medical Oncology, Breast Oncology Center, Cancer Institute Hospital, Japanese Foundation for Cancer Research, Tokyo, Japan; 11 Department of Breast Oncology, International Medical Center, Saitama Medical University, Saitama, Japan

**Keywords:** Chemotherapy-induced nausea and vomiting, Breast cancer, Antiemetics, Guideline

## Abstract

**Purpose:**

To explore the actual status of chemotherapy-induced nausea and vomiting (CINV) through a multicenter prospective cohort study.

**Methods:**

Patients with breast cancer treated with moderately emetogenic (MEC) or highly emetogenic (HEC) chemotherapy were eligible. A 7-day diary was provided for all patients. Acute and delayed CINV were defined as nausea and vomiting that developed ≤ 24 or > 24 h after the start of chemotherapy, respectively. The severity of nausea was evaluated with a visual analog scale (VAS). We also assessed the accuracy of estimations of CINV by medical staff.

**Results:**

In total, 426 patients were included; 352 patients (82.6%) received HEC, and 74 (17.3%) received MEC. In the acute phase, 44.9% of patients receiving HEC and 5.4% receiving MEC experienced nausea, and 12.8% receiving HEC and none receiving MEC experienced vomiting. More patients experienced nausea in both groups and vomiting in MEC during the delayed phase (nausea: 59.4% in HEC and 44.6% in MEC group; vomiting: 11.1% in HEC; and 13.5% in MEC group) than during the acute phase. Estimations of CINV by medical staff were not accurate, with a kappa coefficient of 0.10 and 0.08 for acute nausea and vomiting and 0.02 and 0.01 for delayed. The VAS scores showed that in the HEC group, the degree of nausea was worst on the first day.

**Conclusions:**

The degree of nausea was worst in the acute phase, although delayed nausea was more in proportion in HEC. Estimation by medical staff is not accurate.

## Introduction

Breast cancer is one of the most common cancers worldwide [1, 2]. Survival of patients with breast cancer has dramatically improved because of effective screening system and advances in multimodal therapy, including surgery, radiotherapy, and systemic therapy [3]. Chemotherapy currently plays an important role in both the perioperative period and metastatic setting.

Chemotherapy-induced nausea and vomiting (CINV) is one of the most common and clinically serious adverse reactions to chemotherapy, despite the development of global and domestic guidelines for CINV by the American Society of Clinical Oncology (ASCO) [4], Multinational Association of Supportive Care in Cancer (MASCC)/European Society of Medical Oncology (ESMO) [5], National Comprehensive Cancer Network (NCCN) [6], and Japanese Society of Clinical Oncology (JSCO). These guidelines consistently recommend the combination of a 5-hydroxytryptamine receptor antagonist (5HT3RA) and dexamethasone for moderately emetogenic chemotherapy (MEC), and a combination of three antiemetics comprising the above two drugs and an aprepitant or fosaprepitant for highly emetogenic chemotherapy (HEC). However, the actual status of clinical practice with respect to these guidelines as well as the proportion and degree of CINV under the optimal antiemetics according to the guidelines are not well studied. Therefore, we conducted a nationwide survey to study the incidence of CINV and the use of antiemetics in accordance with the above guidelines. We additionally assessed estimations of CINV by medical staff to determine the accuracy of CINV predictions by physicians and other medical staff.

## Materials and methods

### Study design

This was a multicenter prospective cohort study. A nationwide survey of CINV was conducted by the CINV Study Group of Japan. We herein present the results of the breast cancer cohort; the results of the entire cohort have been published elsewhere [7]. We selected university hospitals, cancer centers, and cancer treatment hospitals certified by the Ministry of Health, Labour and Welfare (which should serve as core centers for cancer treatment) to participate in this study.

### Enrollment of patients

The inclusion criteria have been described in detail elsewhere [7]. In brief, patients scheduled to undergo MEC or HEC for first-time treatment of cancer were eligible for the current study. Classification of the emetogenic risk of anticancer drugs was based on the Guidelines for Appropriate Use of Antiemetic Drugs, Version 1, published by JSCO [8] and created based on the NCCN Clinical Practice Guidelines in Oncology—Antiemetics—ver. 4, 2009.

To investigate estimation of CINV by medical staff, the staff members were requested to fill out questionnaires regarding their estimation of the severity of symptoms in the acute and delayed phases of CINV on the same registration form if they thought that their patients would develop CINV. Acute and delayed CINV were defined as nausea and vomiting that developed ≤ 24 or > 24 h after the start of chemotherapy, respectively.

We aimed to register 450 patients with breast cancer, 700 with gastrointestinal cancer, 200 with gynecological cancer, 200 with hematological malignancies, and 450 with lung cancer.

### Patient diary and case reports

A 7-day diary to record CINV prior to commencement of cancer chemotherapy was provided to each patient. In brief, digestive symptoms such as the development and severity of nausea, frequency of vomiting, amount of food intake, number of salvage treatments, and confirmation of hospitalization or an outpatient visit were recorded. Patients were required to make an entry in the diary every day for 7 days from commencement of anticancer MEC or HEC. The severity of nausea and oral intake were assessed with a linear visual analog scale (VAS; with maximum of 100 mm) and a facial scale. The patients were requested to fill in their diaries and send them to the central office in the return envelope provided.

The following patient characteristics were also collected: sex, age, treatment history, use of an anxiolytic drug before administration of the anticancer drug, use of opioids, alcohol intake history, potential risk factors for CINV (history of motion sickness or pregnancy-related vomiting), Eastern Cooperative Oncology Group (ECOG) performance status, complete blood count, blood biochemistry results, cancer chemotherapy regimen, and details of antiemetic therapy and salvage treatment for CINV.

### Estimation of CINV by medical staff

Medical staff estimated whether the patient would develop nausea, vomiting, and anorexia in both acute and delayed phase prior to chemotherapy, mainly based on the patient characteristics such as age, motion sickness, and experience of hyperemesis gravidarum. And if the medical staff suspected the patient would develop nausea, the degree of nausea was estimated by VAS (scored from 0 to 10).

### Statistical analysis

Differences in the occurrence of CINV between patients receiving HEC or MEC and among risk factors for emesis were analyzed using Fisher’s exact test. The numbers of risk factors for CINV in the collected data sets were analyzed by multivariate logistic regression analysis (Wald’s test). All reported *p* values correspond to a two-sided test, and *p* values of ≤ 0.05 were considered statistically significant. Analyses were carried out with SAS for Windows, release 9.3 (SAS Institute, Cary, NC).

We excluded one patient treated with cisplatin and irinotecan (CDDP + CPT-11) from analysis as this combination regimen is not standard and widely used in ordinary breast cancer.

## Results

### Patients

**Fig. 1:**
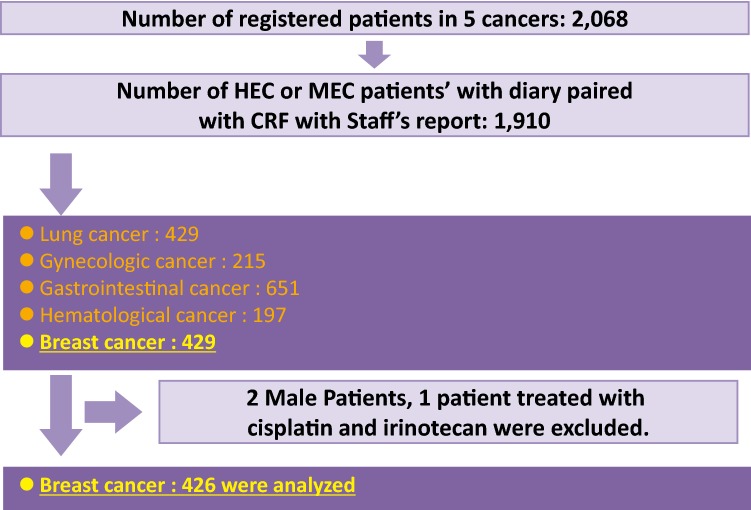
Patient flow. In total, 1910 patients were included in the whole study and 429 had breast cancer. One patient treated with cisplatin and irinotecan and two male patients were excluded; therefore, 426 patients were included for the current analyses

In total, 1910 patients were included between April 2011 and December 2012 in the whole study, and 429 had breast cancer. We excluded one patient treated with cisplatin and irinotecan as this regimen is not used widely for breast cancer. And two male patients were also excluded as our analyses included hyperemesis gravidarum as a covariate (Fig. 1). The characteristics of the 426 patients with breast cancer are listed in Table 1. Almost all of these patients had an ECOG PS of 0. A total of 352 patients (82.6%) received HEC and 74 (17.3%) received MEC. The most common HEC regimen was FEC (5-fluorouracil, epirubicin, and cyclophosphamide), and the most common MEC regimen was TC (docetaxel and cyclophosphamide). In HEC patients, the proportion of adherence to the JSCO guideline was 87% and in MEC 82%.

**Table 1:** Patient characteristics of breast cancer in the study

	Number of patients (*n* = 426)	%
Sex		
Male	0	0.0
Female	426	100.0
Stage		
1	69	16.2
2	241	56.6
3	84	19.7
4	19	4.5
Recurrence	12	2.8
PS		
0	412	96.7
1	13	3.1
2	1	0.2
HEC (*n* = 352)		
FEC	210	59.7
EC	101	28.7
AC	41	11.6
HEC prophylactic regimen		
3 antiemetics	307	87.2
2 antiemetics	32	9.1
Antiemetics other	13	3.7
MEC (*n* = 74)		
TC	62	83.8
CBDCA + DOC	9	12.2
CMF	2	2.7
CBDCA + PTX	1	1.4
MEC prophylactic regimen		
3 antiemetics	3	4.1
2 antiemetics	58	78.4
Antiemetics other	13	17.6

*FEC* fluorouracil + epirubicin + cyclophosphamide, *EC* epirubicin + cyclophosphamide, *AC* doxorubicin + cyclophosphamide, *TC* docetaxel + cyclophosphamide, *CBDCA* carboplatin, *DOC* docetaxel, *CMF* cyclophosphamide + methotrexate + fluorouracil, *PTX* paclitaxel

### Nausea and vomiting

**Fig. 2:**
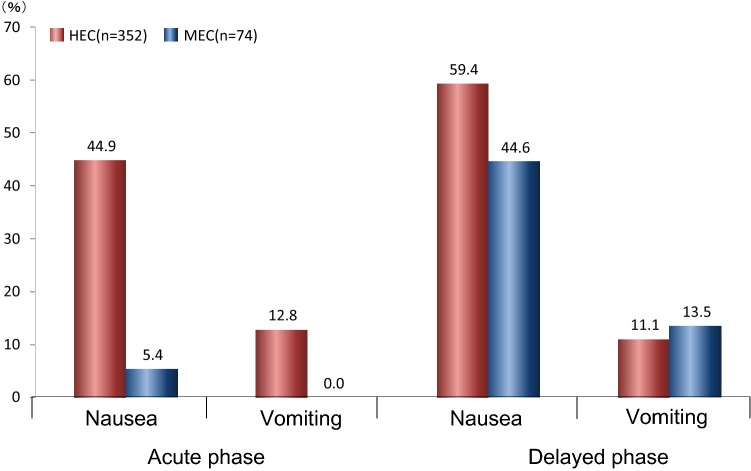
Occurrence of acute/delayed nausea and vomiting in HEC- and MEC-induced CINV. Proportion of patients with nausea and vomiting. Acute phase: ≤ 24 h from administration of emetogenic agents, delayed phase: > 24 h from administration

**Fig. 3:**
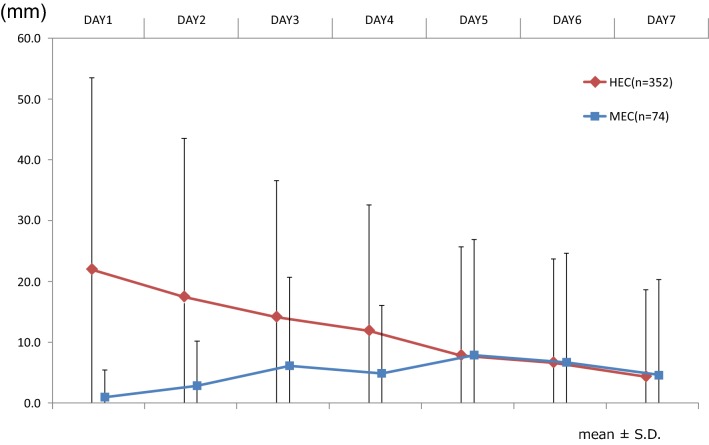
VAS of nausea from day 1 to day 7. Visual analog scale (VAS) for the degree of nausea

Figure 2 shows the proportion of patients with nausea and vomiting. In the acute phase (≤ 24 h from administration of emetogenic agents), 44.9% of patients receiving HEC and 5.4% of patients receiving MEC experienced nausea, and 12.8% of those receiving HEC and 0.0% of those receiving MEC experienced vomiting. In the delayed phase (> 24 h from administration), more patients experienced nausea in both groups and vomiting in MEC during the delayed phase (nausea: 59.4% of those receiving HEC and 44.6% of those receiving MEC; vomiting: 11.1% of those receiving HEC and 13.5% of those receiving MEC) than during the acute phase. The VAS scores (Fig. 3) showed that in patients receiving HEC, the degree of nausea was worst on the first day and gradually improved. Conversely, nausea was sustained without peaking in patients receiving MEC.

### Estimation of nausea and vomiting by medical staff

Table 2 shows the medical staff‘s estimations of whether patients would develop nausea and vomiting. In the acute phase, 78.5% of patients (335 of 426) were estimated to develop nausea; however, 196 patients (46.0%) did not actually experience nausea. The kappa coefficient was 0.0958. With regard to vomiting, the kappa coefficient was 0.0795. In the delayed phase, the kappa coefficient was 0.0237 for nausea and 0.0092 for vomiting.

**Table 2:** Estimation of chemotherapy-induced nausea and vomiting (CINV) by medical staff compared with actual CINV

	Estimation by medical staff (*n* = 426)		
	No CINV	CINV	Total
Acute nausea kappa = 0.0958			
No	68 (16.0%)	196 (46.0%)	264 (62.0%)
Yes	23 (5.4%)	139 (32.6%)	162 (38.0%)
Total	91 (21.4%)	335 (78.6%)	426 (100%)
Acute vomiting kappa = 0.0795			
No	305 (71.8%)	75 (17.6%)	380 (89.4%)
Yes	31 (7.3%)	14 (3.3%)	45 (10.6%)
Total	336 (79.1%)	89 (20.9%)	425 (100%)
Delayed nausea kappa = 0.0237			
No	49 (11.5%)	135 (31.8%)	184 (43.2%)
Yes	59 (13.8%)	183 (43.0%)	242 (56.8%)
Total	108 (25.4%)	318 (74.6%)	426 (100%)
Delayed vomiting kappa = 0.0092			
No	312 (73.2%)	65 (15.3%)	377 (88.5%)
Yes	40 (9.4%)	9 (2.1%)	49 (11.5%)
Total	352 (82.6%)	74 (17.4%)	426 (100%)

*CINV* chemotherapy-induced nausea and vomiting

### Analysis of risk factors for nausea and vomiting

**Fig. 4:**
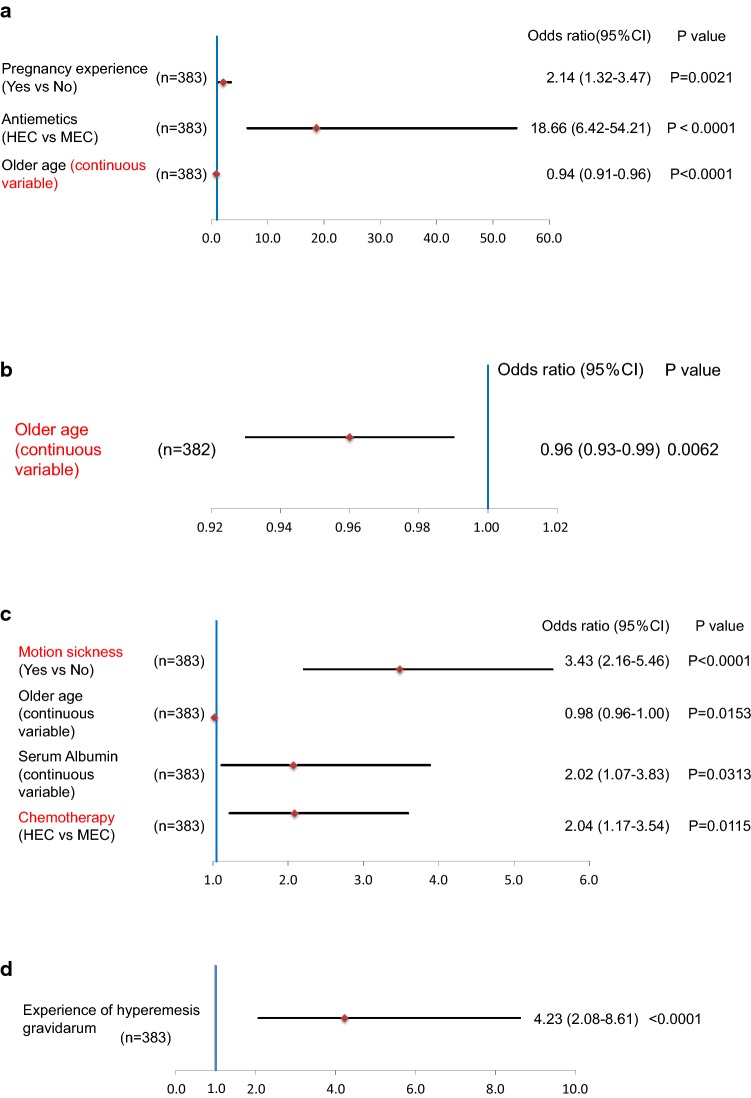
Multivariate analysis of prognostic factors. **a** Multivariate analysis of prognostic factors for acute-phase nausea. Multivariate analysis showed that pregnancy experience and treatment with HEC were significantly correlated with developing acute-phase nausea. **b** Multivariate analysis of prognostic factors for acute-phase vomiting. Younger age was the only factor which was significantly correlated with acute-phase vomiting. **c** Multivariate analysis of prognostic factors for delayed-phase nausea. Experience of motion sickness, younger age, a higher serum albumin concentration, and treatment with HEC were significantly correlated with delayed-phase nausea. **d** Multivariate analysis of prognostic factors for delayed-phase vomiting. Experience of hyperemesis gravidarum was the only factor which was correlated with delayed-phase vomiting. Covariates included in the analyses were as follows; history of motion sickness (yes vs no), reproductive history (yes vs no), history of pregnancy-related vomiting (yes vs no), alcohol intake history (yes vs no), age (continuous variable), hemoglobin (continuous variable), serum albumin concentration (continuous variable), number of prophylactic antiemetics (3 vs 2), use of 5HT3 antagonist (yes vs no), and HEC vs MEC

Multivariate analysis revealed that pregnancy experience (odds ratio [OR] 2.14; 95% confidence interval [CI] 1.32–3.47; *p* = 0.0021), treatment with HEC (odds ratio [OR] 18.7; 95% confidence interval [CI] 6.42–54.21; *p* < 0.0001), and younger age (odds ratio [OR] for older age as continuous variables, 0.94; 95% confidence interval [CI] 0.91–0.96; *p* < 0.0001) were significantly correlated with developing acute-phase nausea (Fig. 4a). Younger age was also significantly correlated with acute-phase vomiting (OR 0.96; 95% CI, 0.93–0.99; *p* = 0.0062) (Fig. 4b).

In the delayed phase, experience of motion sickness (OR 3.43; 95% CI 2.16–5.46; *p* < 0.0001), younger age (OR 0.98; 95% CI 0.96–1.00; *p* = 0.0153), a higher serum albumin concentration (OR 2.02; 95% CI 1.07–3.83; *p* = 0.0313), and treatment with HEC (odds ratio [OR] 2.04; 95% confidence interval [CI] 1.17–3.54; *p* = 0.0115) were significantly correlated with nausea (Fig. 4c). Experience of hyperemesis gravidarum was also correlated with vomiting (OR 4.23; 95% CI 2.08–8.61; *p* < 0.0001) (Fig. 4d).

## Discussion

Our study has demonstrated the actual status of CINV in patients with breast cancer in accordance with the current guidelines in Japan. Although the Hawthorne effect may have been present in this study (i.e., the treating physician might have paid more attention than in routine daily practice), high adherence to the guidelines deserves special mention. In the INSPIRE study conducted in the United States, only 57.3% of patients were treated with guideline-consistent antiemetic therapy, although adherence to antiemetic guidelines can significantly reduce the incidence of CINV after HEC and MEC [9]. In the PEER study conducted in Europe, guideline-consistent antiemetic therapy was administered to 55% and 46% of patients during the acute and delayed phases, respectively, and to 29% of patients throughout the overall study period (acute plus delayed phases); this was true despite the fact that again, adherence to antiemetic guidelines can significantly reduce the incidence of CINV [10].

Grunberg et al. reported that physicians and nurses markedly underestimated the incidence of delayed nausea and emesis after both HEC and MEC [11]. Our study consistently confirmed that it is difficult to accurately estimate whether a patient will develop CINV after administration of HEC or MEC. Therefore, we recommend to adhere to the current guidelines and not to reduce antiemetics. Because CINV is one of the most clinically serious adverse reactions to chemotherapy, accurate estimation whether the patient will develop CINV or not is crucial; however, we found no sufficient predictors of CINV despite the fact that our study confirmed the previously reported risk factors for CINV. We found that younger age was a consistent risk factor for acute nausea, acute vomiting, and delayed nausea, and these findings are consistent with those of a previous study [12].

The proportion of patients who experienced nausea was higher in the delayed phase, which is consistent with a previous study [9–12]. However, the degree of the nausea was worst on the first day based on the VAS score. This is important information, because almost all recent investigations to improve the control of CINV have focused on delayed nausea and vomiting; in the present study, however, the patients suffered most from acute nausea, not delayed nausea. In future trials, we should evaluate VAS scores and be particularly aware of the degree of acute nausea.

In conclusion, the present study demonstrated high compliance to the CINV guidelines. Although underestimation of CINV by treating staff was resolved to some extent, accurately estimating CINV is still difficult. VAS scores revealed that acute nausea remains a clinically serious adverse reaction to HEC.
